# Influences of Ultrafine Ti(C, N) on the Sintering Process and Mechanical Properties of Micron Ti(C, N)-Based Cermets

**DOI:** 10.3390/ma16083175

**Published:** 2023-04-18

**Authors:** Lili Ma, Zaiyang Zhao, Yurong Wu, Jingjing Sun, Siyong Gu, Houan Zhang

**Affiliations:** 1 Fujian Province Key Laboratory of Functional Materials and Applications, Xiamen University of Technology, Xiamen 361024, China; z279160894@163.com (Z.Z.); 2014113201@xmut.edu.cn (Y.W.); 2015000093@xmut.edu.cn (J.S.); 2010110508@xmut.edu.cn (S.G.); 2 School of Materials Science and Engineering, Xiamen University of Technology, Xiamen 361024, China

**Keywords:** ultrafine, micron Ti(C, N)-based cermets, solid-state sintering stage, phase evolution, microstructure, mechanical properties

## Abstract

For investigating the influence mechanism underlying ultrafine Ti(C, N) within micron Ti(C, N)-based cermets, three cermets including diverse ultrafine Ti(C, N) contents were employed. In addition, for the prepared cermets, their sintering process, microstructure, and mechanical properties were systematically studied. According to our findings, adding ultrafine Ti(C, N) primarily affects the densification and shrinkage behavior in the solid-state sintering stage. Additionally, material-phase and microstructure evolution were investigated under the solid-state stage from 800 to 1300 °C. Adding ultrafine Ti(C, N) enhanced the diffusion and dissolution behavior of the secondary carbide (Mo_2_C, WC, and (Ta, Nb)C) under a lower sintering temperature of 1200 °C. Further, as sintering temperature increased, adding ultrafine Ti(C, N) enhanced heavy element transformation behaviors in the binder phase and accelerated solid-solution (Ti, Me) (C, N) phase formation. When the addition of ultrafine Ti(C, N) reached 40 wt%, the binder phase had increased its liquefying speed. Moreover, the cermet containing 40 wt% ultrafine Ti(C, N) displayed superb mechanical performances.

## 1. Introduction

Ti(C, N)-based cermets demonstrate great wear resistance, excellent hardness, and chemical stability, and as a result, they have been extensively adopted as high-speed cutting tools [1,2,3]. Nonetheless, the balance between cermet strength and toughness is always poor compared with a WC-Co system, and this has dramatically restricted their use [4,5].

As for Ti(C, N)-based cermets, their fracture toughness can be primarily decided by ductile metallic binder-phase content and ceramic-phase grain size. It has been reported that using submicron-/nanosized WC powders within hard metals significantly enhances mechanical performance [6]. Previous reports suggest that using submicron- or nanosized hard-phase powders as the raw material instead of microsized Ti(C, N) efficiently enhances cermet hardness [4,7,8,9]. However, this hardness enhancement, consisting of reducing the size of raw materials, offers toughness to some degree [10]. In addition, to obtain the synergistic effect of different Ti(C, N) sizes on cermet mechanical performances, ultrafine [11] nanosized [12] Ti(C, N) was introduced into micro-Ti(C, N)-based cermets or coarse Ti(C, N) [13] was introduced into ultrafine Ti(C, N)-based cermets. However, there is still a lack of understanding of the effects of ultrafine or nanosized Ti(C, N) powder on the sintering and densification process for micro-Ti(C, N)-based cermets.

Consequently, the carbon thermal reduction-nitridation process was employed for fabricating ultrafine Ti(C, N) powders. Further, the produced ultrafine Ti(C, N) powders, along with commercial microsized Ti(C, N) powders, were applied as the raw material in the preparation of Ti(C, N)-based cermets using a traditional powder metallurgic approach. This study examined how ultrafine Ti(C, N) affected microstructure as well as mechanical performance. Additionally, the role of ultrafine Ti(C, N) powder in the cermet microstructure evolution process and phase formation, together with shrinkage characteristics, were demonstrated.

## 2. Experimental Procedure

Raw materials included self-made ultrafine Ti(C, N) powders (200~500 nm) and commercially available microsized Ti(C, N) (mean size, 1.5 µm), WC (mean size, 1.2 µm), Mo_2_C (mean size, 2.0 µm), (Ta, Nb)C (8:2) (mean size, 2.0 µm), Ni (mean size, 1.5 µm) and Co (mean size, 0.8 µm) powders. Table 1 displays the predetermined cermet compositions introduced, with increasing ultrafine Ti(C, N) contents. Typically, the ultrafine Ti(C, N) content of the Ti(C, N) powders was 0 wt%, 20 wt%, and 40 wt%, respectively.

**Figure 1:**
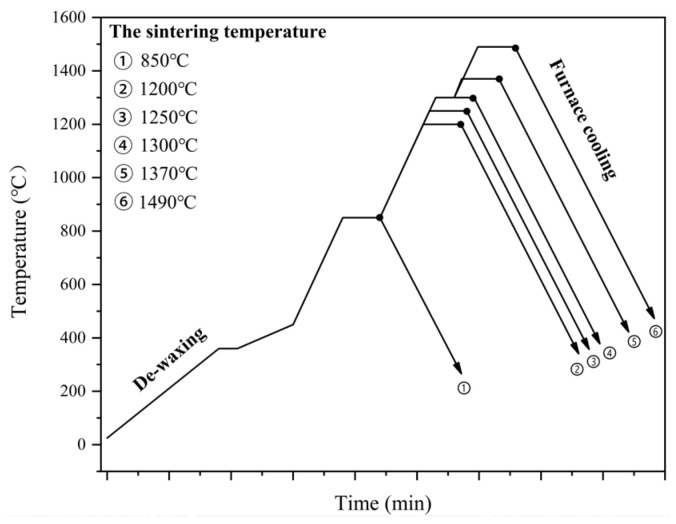
Sintering procedure for Ti(C, N)-based cermets under vacuum.

To prepare cermets, 6 mm WC-10Co alloy balls were utilized as the milling balls, followed by mixing using starting powders at a 7:1 ball-to-powder weight ratio within pure water, and then milling for 72 h under room temperature. After spray drying, the powder mixture was later compacted into standard sample strips (dimension, 25 mm × 8 mm × 6.5 mm) under 250 MPa. Next, we sintered the Ti(C, N)-based samples in identical experimental conditions. During sintering, a ~10Pa vacuum pressure was maintained. To study the microstructure and phase transition, the sintering procedure was conducted under diverse temperatures such as 850, 1200, 1250, 1300, 1370, and 1490 °C, and the samples were allowed to stand for 60 min (the sintering schedules are presented in Figure 1). Finally, the samples sintered in 1490 °C for 60 min were utilized for detecting the microstructure as well as mechanical performance.

With regard to sintered cermets, their densities were determined with Archimedes approach. Considering the incomplete sample density sintered under decreased temperatures, lower temperature sample densities were determined based on an average of five values through calculating the sample mass ratio to the volume.

X-ray diffraction (XRD) (Smartlab 3 KW, Rigaku, Japan) was conducted for analyzing phases using Cu Kα radiation at the 0.02°/s step rate. Meanwhile, MDI JADE (Edition 6.5, Materials data Ltd., America) was employed for the numerical analysis of XRD patterns. Scanning electron microscopy (SEM) (Sigma 500, ZEISS, Jena, Germany) coupled with EDXA was performed for characterizing the morphology of raw materials and the sintered cermets specimens. TEM and HRTEM were performed on a Talos f200s instrument at the 200 KV (FEI) accelerating voltage. A universal material testing machine (CMT5105, SANS, China) was applied in measuring transverse rupture strength (TRS) with the three-point bending approach (span 20 mm, crosshead speed 0.5 mm/min). In addition, Vickers-hardness (HV) was measured at the 30 kg indenter load onto the polished cermet surface, whereas fracture toughness (K _IC_) was determined using an equation put forward by Shetty et al. [14]. Mechanical performance data represent the means of six tests.

## 3. Results and Discussion

### 3.1. Ultrafine TiCN Powders

**Figure 2:**
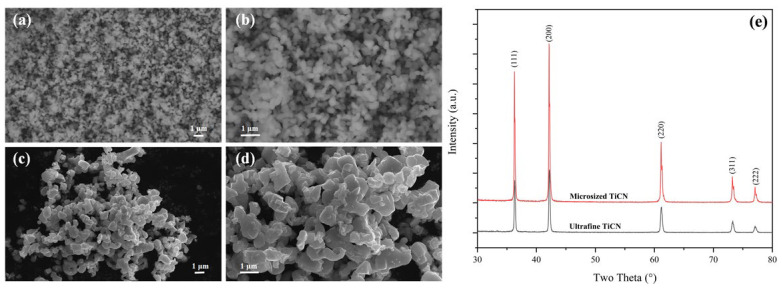
SEM images and XRD patterns of the self-prepared ultrafine and commercial microsized TiCN powders ((**a**,**b**) display SEM images for ultrafine TiCN powder, (**c**,**d**) are the SEM images of microsized TiCN powder, (**e**) is the XRD patterns of these two kinds of powders).

In this paper, ultrafine Ti(C, N) powders were synthesized through a carbothermal reduction-nitridation reaction. Ultrafine anatase (~500 nm) and nano-amorphous carbon black (~100 nm) were adopted as starting materials. The ultrafine Ti(C, N) with oxygen as low as 0.62 wt% was prepared by lowering the sintering temperature from over 1500 °C to 1400 °C. From Figure 2a,b, we can observe that the prepared ultrafine Ti(C, N) has a relatively uniform size of around 200 nm. However, the particle size of the commercially available Ti(C, N) is about 1.0–2.0 um (Figure 2c,d), with oxygen content as high as 0.52 wt%.

Figure 2e exhibits the XRD patterns for two kinds of Ti(C, N) powder. Obviously, there is only one kind of phase in them—Ti(C, N). The identical peak positions of these two kinds of powders indicates their similar Ct % and N%. However, the ultrafine Ti(C, N) has a bigger FWHM compared to the microsized Ti(C, N). In line with the Scherrer equation (D = Kγ/Bcosθ), the crystallite size of ultrafine Ti(C, N) is only 49.4 nm, which is far less than that of the microsized Ti(C, N) at 87.3 nm.

### 3.2. Function of Ultrafine Ti(C, N) in Densification Behaviors of TiCN-Based Cermets

**Figure 3:**
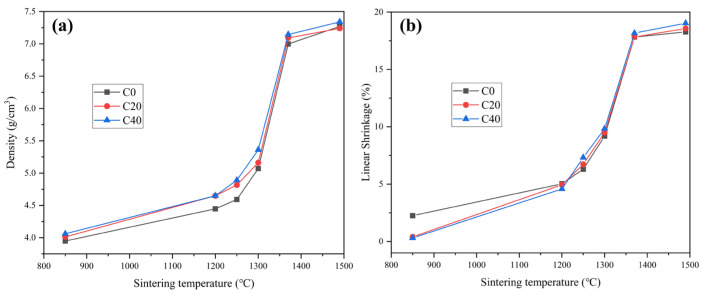
Density (**a**) and Linear shrinkage (**b**) versus sintering temperature of cermets specimens.

Figure 3 displays the density and linear shrinkage of all three sintered cermet specimens in a vacuum at different temperatures. It is well known that the sintering process of the cermets was divided into solid and liquid states. In Figure 3, the sample’s density and linear shrinkage display a slow increase within the early temperature range of 850~1300 °C, and later a substantial elevation after 1300 °C. This means that the system was in the solid-state sintering stage before 1300 °C, then converted to the liquid-state sintering stage at the >1300 °C sintering temperature [15,16].

Compared to the cermets’ density and linear shrinkage curves, when temperatures were above 1300 °C, C0, C20, and C40 displayed similar sharp densification behavior. Figure 3 shows that at 1490 °C, the three samples exhibit a similar ultimate linear shrinkage (18.27% for C0, 18.57% for C20, and 19.00% for C40) with almost consistent density (7.27 g/cm^3^ for C0, 7.24 g/cm^3^ for C20 and 7.32 g/cm^3^ for C40). However, as for sintering temperature <1300 °C, a difference between three cermets is quite apparent. As seen in Figure 3a, the densification rate of the cermets was accelerated after adding ultrafine Ti(C, N). Firstly, The sample densities at 1200 °C and 1250 °C were enhanced along with the added 20 wt% ultrafine Ti(C, N). As ultrafine Ti(C, N) content further elevated to 40 wt%, the densities of C40 at 1250 °C and 1300 °C were further enhanced to 4.89 g/cm^3^ and 5.36 g/cm^3^, leading to a steeper density curve from 1200 °C to 1300 °C. Meanwhile, the linear shrinkage curve changes were similar to those of the density curve (Figure 3b). The linear shrinkage under 1200 °C was enlarged immediately when 20 wt% ultrafine Ti(C, N) was added. Further, as sintering temperature elevated, C20 had slightly elevated linear shrinkage compared with C0. As well as this, as ultrafine Ti(C, N) content further increased to 40 wt%, the linear shrinkages at 1250 °C and 1300 °C were increased, resulting in an increase in the slope of the linear shrinkage curve from 1200 °C to 1300 °C.

So, adding ultrafine Ti(C, N) powder significantly accelerates Ti(C, N)-based cermet sintering in the solid-state, as observed from the submicron Ti(C, N) cermet system [4]. Nonetheless, there are few systematical articles about how ultrafine Ti(C, N) powder affects Ti(C, N)-based cermets in terms of their evolution behavior during solid-state sintering.

### 3.3. Function of Ultrafine TiCN in Microstructure and Phase Evolution during the Solid-State Stage

Some studies have reported the Ti(C, N) cermet sintering process [16,17,18,19]. This study also examined the function of ultrafine Ti(C, N) in the solid-state sintering process.

**Figure 4:**
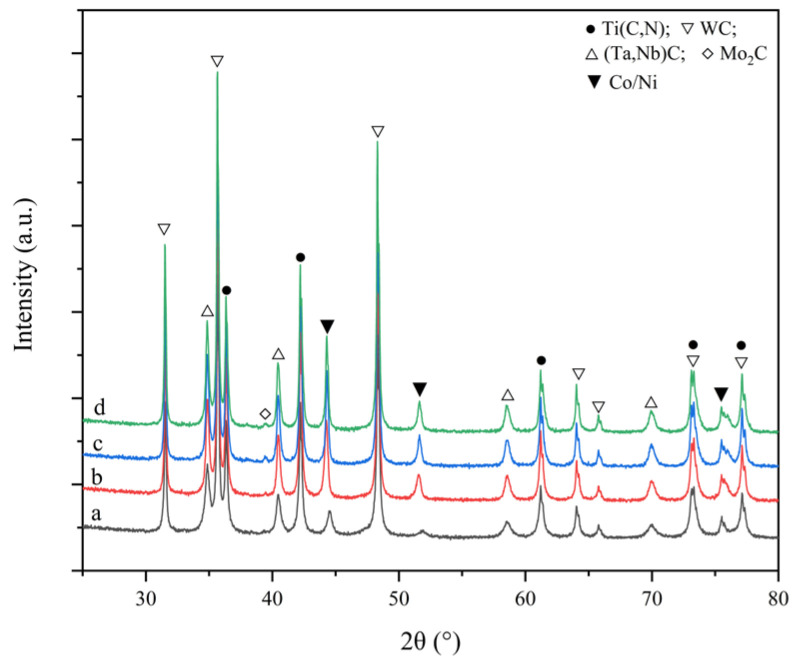
XRD patterns for raw powder mixture. (a: C0 at atmospheric temperature; b: C0 at 850; c: C20 at 850; d: C40 at 850).

It has been confirmed many times that at a sintering temperature of <850 °C [16] or even <900 °C [17,18], the microstructure and phase composition of the cermets had no apparent changes compared with the raw mixed powders. Similarly, at the >850 °C sintering temperature, the phases, along with the phase content, of all three samples were almost the same as the raw mixed powders, as shown in Figure 4. The discussion about the solid-state sintering process covers the temperature range of 1200–1300 °C as metallurgy reaction under 1000 °C is still very limited [20].

**Figure 5:**
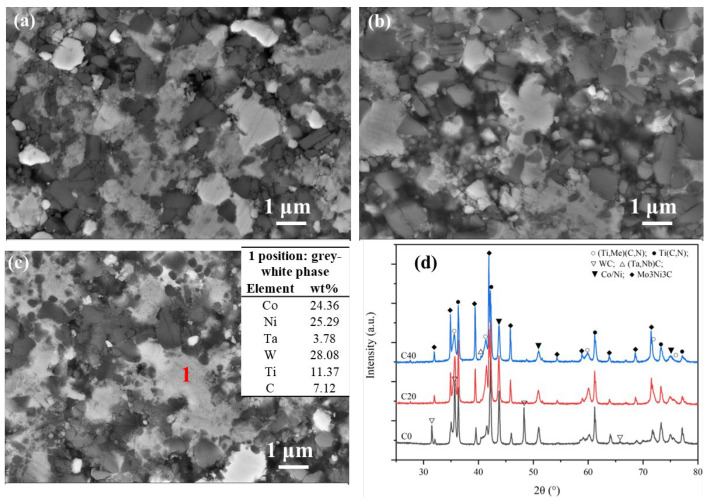
The SEM micrographs ((**a**) C0, (**b**) C20, (**c**) C40) and XRD patterns (**d**) of all three cermets sintered in vacuum under 1200 °C. The table in figure (**c**) displays the EDS result at the position 1.

Figure 5 exhibits the morphology and XRD patterns for all three cermets sintered at 1200 °C. As seen in Figure 5a, the solid-phase diffusion behavior is noticeable. The grey binder phase started agglomerating and presented as a grey-white phase, indicating that the heavy element (W/Mo/Ta) diffused into the binder phase at this temperature (the table in Figure 5c displays the EDS results). After adding ultrafine TiCN, there was a viscous grey-white phase along with an increasing number of ultrafine Ti(C, N) particles wrapped up by the viscous grey-white phase, as shown in Figure 4b,c. This means that the diffuse or bonded reaction between the secondary carbide (WC, Mo_2_C, (Ta, Nb)C) with the bind and the Ti(C, N) is evident with the ultrafine Ti(C, N) powders under 1200 °C.

In addition, Figure 5d shows that the diffraction peaks of hexagonal WC decreased significantly for C0, whereas those peaks disappeared for C20 and C40. This means that hexagonal WC had been consumed by dissolution within the binder phase or Ti(C, N), and ultrafine Ti(C, N) increased its dissolution rate [21]. As W or Mo was dissolved in the binder, the M_6_C (Mo_3_Ni_3_C) appeared in C0. Compared to C0, the diffraction intensity of M_6_C in C20 and C40 increased significantly, suggesting that more secondary carbide had been consumed and converted to the M_6_C phase. As well as this, the appearance of the solid-solution phase showing diffraction peaks of (Ti, Me)(C, N) (Me: W, Mo Ta, Nb heavy element) in C20 and C40 also demonstrates the role of ultrafine Ti(C, N) in advancing the solid-solution reaction between secondary carbides (WC, Mo_2_C or (Ta, Nb)C) and Ti(C, N) [18,22]. So, the addition of ultrafine TiCN not only shortened the diffusion distance between the secondary carbide and binder and accelerated the formation of the carbon defect-phase M_6_C, but it also benefited the solid-solution reaction. However, the weak diffraction peaks of TaC/NbC mean there is still undissolved TaC or NbC in all three cermets.

**Figure 6:**
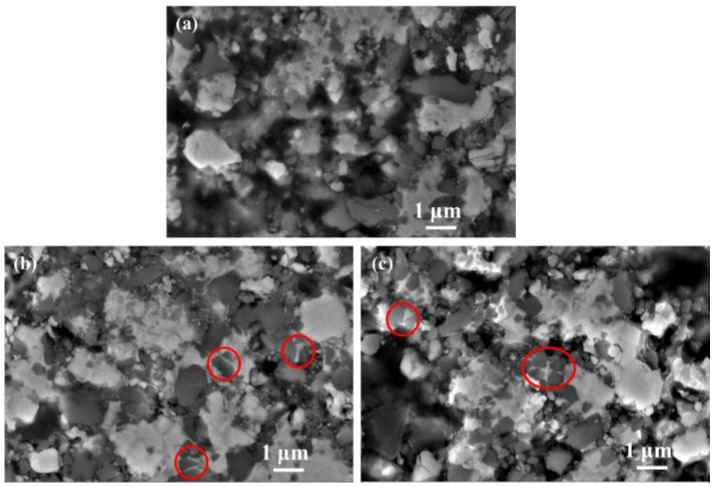
The SEM micrographs of (**a**) C0, (**b**) C20, (**c**) C 40 cermets sintered in a vacuum at 1250 °C.

**Figure 7:**
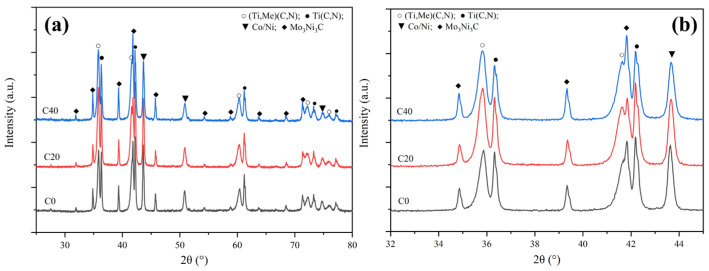
(**a**) XRD patterns for three cermets sintered under 1250 °C; (**b**) enlarged XRD patterns ranging from 32° to 45°.

Figure 6 displays the microstructures of all three cermets sintered at 1250 °C. Although the binder phase has not yet entered the liquid phase, the capillary effect is displayed in Figure 6b,c, indicated by red circles where the larger Ti(C, N) particles were rearranged and compacted under the force of the binder phase. The hard carbide particles were rearranged under the capillary force of the viscous binder in C20 and C40, and more densification behavior was seen, especially for C40. Nevertheless, the microstructure of C0 is similar to C20 at 1200 °C. Moreover, the XRD patterns for cermets indicate that second carbides disappear after reaching the 1250 °C sintering temperature. Diffraction peaks of (Ti, Me)(C, N) and M_6_C increased significantly at the same time, as shown in Figure 7a. Noteworthily, for the Ti(C, N) phase, its diffraction peak in C40 cermets shows a higher-angle shift compared to C0 and C20 in the enlarged XRD patterns, as shown in Figure 7b, suggesting that C content within Ti(C, N) of C40 was less than in C20 and C0. Additionally, Ti(C, N) (~25 wt%) content within C40 was also lower than in C20 (~28.5 wt%) and C0 (~33.7 wt%). This demonstrated the diffusion and reaction of the WC, Mo_2_C, and (Ta, Nb)C with Ti(C, N) or the binder phase, which Li et al. [18] observed as being accelerated by ultrafine Ti(C, N) together with the generated (Ti, Me)CN solid-solution phase. Due to the higher affinity between the secondary carbide and binder for [C] [19,21,22], the TiCN with higher [C] content reacts or dissolves first.

**Figure 8:**
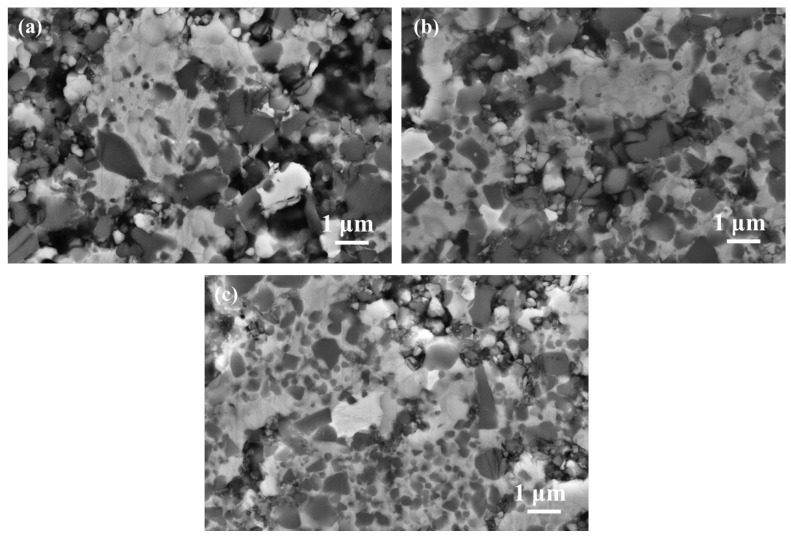
SEM images for (**a**) C0; (**b**) C20 and (**c**) C40 sintered at 1300 °C.

At the 1300 °C sintering temperature, the binder had not yet completely liquated, the capillary effect of the binder was further strengthened, and several ceramic particles were bonded by the white-grey binder phase (Figure 8a). However, the binder-phase distribution was more uniform in C40 than in C20 and C0. From Figure 8c, it seems that the densification behavior was improved by the extensive liquid phase, and large residual pores came from the inadequate sintering temperature. So, the finer Ti(C, N) advanced the solid-state sintering process, encouraging the binder phase’s liquefying speed.

**Figure 9:**
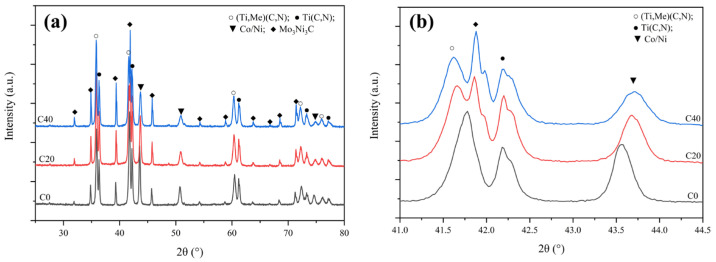
The XRD patterns for three cermets fabricated under the sintering temperature of 1300 °C: (**a**) Full spectrum; (**b**) enlarged spectrum.

Figure 9 shows the XRD patterns for cermet specimens subjected to sintering under 1300 °C (Figure 9), and only the (Ti. Me)(C,N), M_6_C, Ti(C, N), and Co/Ni binder phases exist. Moreover, (Ti, Me)(C,N) and M_6_C contents were increased from 25 wt% and 10.2 wt% for C0 to 31 wt% and 16 wt% for C40. Notably, the diffraction intensity of the binder phase in C0 had the biggest lattice parameter at 3.596 Å (Table 2), which indicated that the heavy elements (W, Ta, Nb, and Mo) were almost dissolved into the binder phase in C0. However, they precipitated from the binder and formed into solid-solution phases in C20 and C40. As the binder phase is believed to act as an essential transport medium for W, Ti, Mo, Ta, Nb, C, and N from secondary carbides or Ti(C, N) into solid solution [17], adding ultrafine Ti(C, N) within C20 and C40 promoted the diffusion, dissolution, and precipitation of secondary carbides.

### 3.4. Role of Ultrafine TiCN in Cermet Morphology and Mechanical Performance

**Figure 10:**
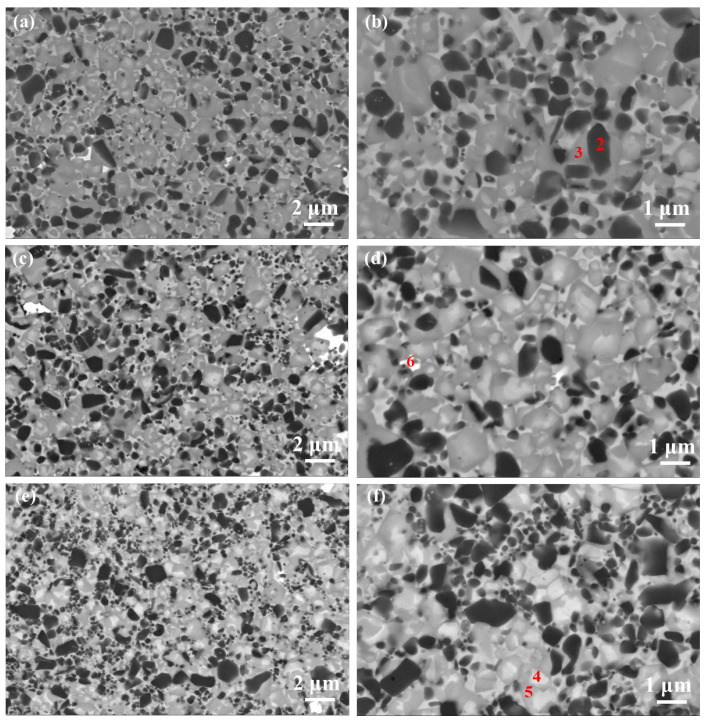
The SEM images showing the prepared cermets under 1490 °C: (**a**,**b**) C0; (**c**,**d**) C20, (**e**,**f**) C40.

Figure 10 shows SEM images for Ti(C, N)-based cermets with different amounts of ultrafine Ti(C, N) addition. Figure 10 verifies the core-rim structure, and there are two rim types, including white core-gray and black core-white/-gray rims. The EDS analysis results of different phases in Figure 10 are displayed in Table 3. Because of the rapider reaction between ultrafine Ti(C, N) and WC as well as (Ta, Nb)C for the formation of (Ti, Me)(C,N) solid solution [4], white core-grey rims had an enhanced proportion after adding ultrafine Ti(C, N) (Figure 10c–f). In addition, the uniform dispersion of binders that contained part of the rimless black grains was seen. Similarly, the number of rimless black grains increased with elevated ultrafine Ti(C, N) content, as the rimless black grain was ultrafine Ti(C, N) residue [23]. Moreover, the fourth predominantly white secondary phase [24] (or eta phase), due to the excessive W and Ta reaction with the binder phase, was observed in all three cermets, which their XRD patterns can also prove. 

**Figure 11:**
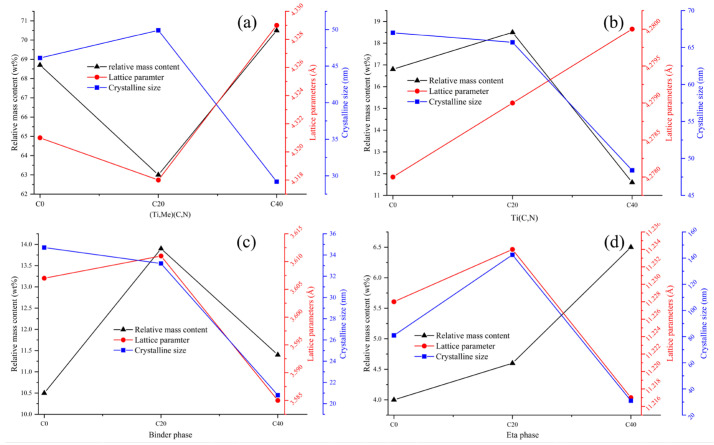
The lattice parameters (Å), the relative mass content (wt%), and crystalline size (nm) of different phases according to the XRD patterns for the three cermets: (**a**) for (Ti, Me)(C, N), (**b**) for Ti(C, N), (**c**) for the binder phase and (**d**) for the eta phase.

Figure 11 displays the lattice parameters, relative mass content, and crystalline size of the phases according to the XRD patterns of the three cermets. It is apparent that the cermets have four different phases, which is consistent with the SEM morphologies. Compared to the other two cermets, the solid solution (Ti, Me) (C, N) has largest content, and Ti(C, N) has lowest content in C40. Additionally, the binder phase in C40 has the smallest lattice parameters, which indicates that there are fewer heavy elements (W, Ta, etc.) in the binder. As well as this, the crystalline size of the four phases in C40 is also the smallest compared to the other two samples.

It is well known that the microstructure substantially impacts mechanical properties. Table 4 shows that as the ratio of ultrafine/microsized Ti(C, N) increases, the hardness of the cermet decreases first before increasing slightly. This may be attributed to Hall–Peth effect of the hard phase in C40 due to the small crystalline size. However, the highest binder-phase content in C20 compromises its hardness a little bit. It has been reported that the hardness of the cermet is enhanced through adding ultrafine Ti(C, N); however, the binder phase was harmful for hardness. In addition, a large amount of homogenized (Ti, Me)CN and secondary white phases were favorable for K _IC_ as well as TRS [24]. C40 had the highest K _IC_ (8.0 MP•m^1/2^) and TRS (1440 MPa) among the three cermets.

**Figure 12:**
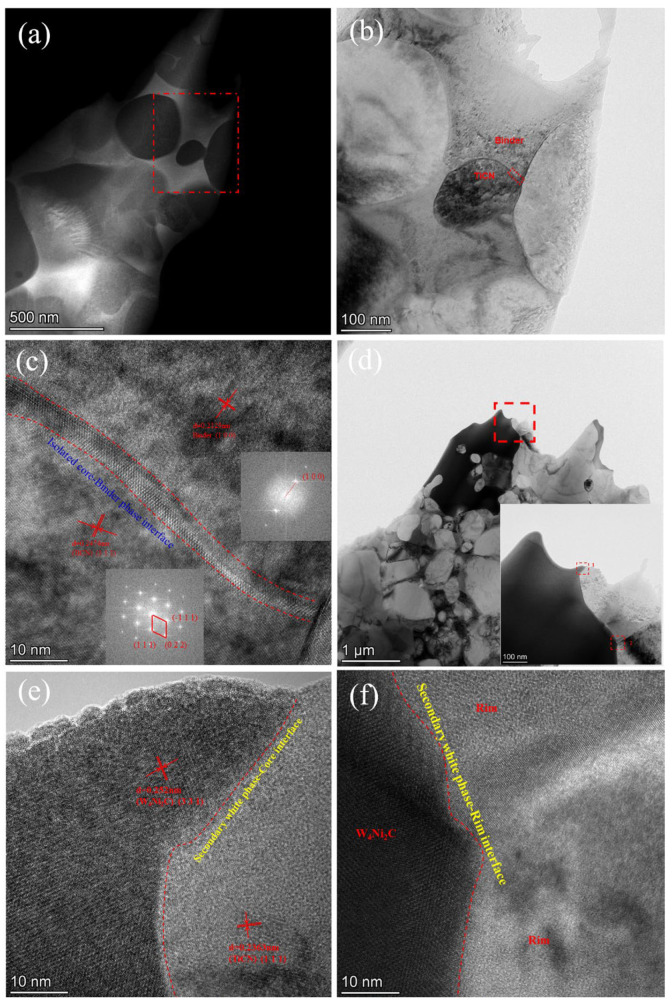
(**a**)The TEM image showing C40 prepared under the sintering temperature of 1490 °C, (**b**) magnified view of red box from (**a**), (**c**) the HRTEM image of the red dotted region in (**b**), (**d**) TEM image of the secondary white phase, (**e**,**f**) are the HRTEM graphs of selection 1 and 2 of (**d**).

To obtain more information on the secondary white phase and the isolated black core, TEM and HRTEM images were captured, and the results are shown in Figure 12. The semi-coherency state at the rimless black bore-binder interface is captured in Figure 12c, which displays the strong bonding strength between the binder phase and rimless core. Figure 12e and Figure 12f exhibits the interface states of the secondary white phase and black core or grey rim. The results show a strong interaction between the secondary white phase and core/rim structure, indicating that the tiny and dispersed white particles will not destroy the mechanical property of the cermets.

## 4. Conclusions

The role of adding ultrafine Ti(C, N) in morphology and phase evolution has been discussed in this study. The following conclusions were obtained:

(1) Adding ultrafine Ti(C, N) accelerates the cermets’ shrinkage rate, especially for the solid-state sintering process.
(2) Adding ultrafine Ti(C, N) improves the diffusion of heavy elements such as W, Ta, Nb into the binder phase and Ti(C, N) at 1200 °C. As the sintering temperature further elevates, the heavy-element (W, Mo Ta, and Nb) transfer behavior from the binder phase is enhanced effectively to form the solid-solution (Ti, Me)CN phase through ultrafine Ti(C, N), advancing phase and morphology evolution.
(3) Adding ultrafine Ti(C, N) also benefits microstructure and mechanical performance, which provides a solution for achieving cermets with excellent mechanical performance.

## Figures and Tables

**Table 1:** Nominal composition of each cermets specimen (wt%).

Cermets	Microsized-TiCN	Ultrafine-TiCN	WC	Mo_2_C	TaNbC	Co	Ni
C0	50	0	25	1	10	7	7
C20	40	10	25	1	10	7	7
C40	30	20	25	1	10	7	7

**Table 2:** Lattice parameters for solid-solution (Ti, Me)CN and TiCN and Co/Ni binder phases within the cermets prepared under the sintering temperature of 1300 °C.

	(Ti, Me) CN (Å)	TiCN (Å)	Co/Ni (Å)
C0	4.329	4.278	3.596
C20	4.337	4.279	3.585
C40	4.338	4.277	3.580

**Table 3:** EDS analyses of different phases marked by spot 2, 3, 4, 5 and 6 in Figure 10.

Element	wt.%				
	2 Black Core	3 Grey Rim around Black Core	4 White Core	5 Grey Rim around White Core	6 Eta Phase
Co	0.68	0.26	0.53	1.17	10.34
Ni	0.52	0.05	0.56	0.89	6.25
Ta	3.48	9.30	12.50	12.27	0.59
W	6.98	20.70	32.13	24.57	72.07
Ti	62.14	43.65	23.27	34.15	1.90
Mo	0.05	0.79	1.98	0.23	1.60
Nb	0.50	2.88	3.99	3.01	0.16
C	17.98	18.61	23.93	21.11	6.39
N	7.67	3.68	1.12	2.57	0.69

**Table 4:** Mechanical performances (hardness (HV30), fracture toughness (K _IC_), together with transverse rupture strength (TRS)) of cermets.

Cermet	HV30	K _IC_/MPa·m^1/2^	TRS/MPa
C0	1530	7.86	1180
C20	1520	7.80	1140
C40	1540	8.00	1440

## Data Availability

The data utilized in the present work can be obtained from this article.
